# Supplementary material for: COVID-19 symptom severity and duration among outpatients, July 2021-May 2023: The PROTECT observational study
Source: PLoS One. 2025 Feb 21;20(2):e0314518. doi: 10.1371/journal.pone.0314518 (PMC11844841; doi:10.1371/journal.pone.0314518)
Supplement: S1 Table — (DOCX) [file pone.0314518.s001.docx]

|  | **Coefficient (95% Confidence Interval)** | **p-value** |
| --- | --- | --- |
| **sex [Male]** | 0.4816579 (0.1838150, 1.228562) | 0.129 |
| **age [>=45]** | 0.2447548 (0.07650320, 0.7005155) | 0.0116 |
| **race [Black or African American]** | 2.047749 (0.3556913, 16.67283) | 0.447 |
| **race [Asian]** | 0.8392855 (0.1293674, 6.217699) | 0.8561 |
| **race [Other]** | 0.3525757 (0.05484309, 2.357371) | 0.2647 |
| **vaccination [2 or more doses]** | 1.069501 (0.1084698, 7.530355) | 0.9483 |
| **comorbidities [1]** | 0.4004678 (0.08128124, 1.670057) | 0.2256 |
| **comorbidities [2]** | 2.324588 (0.5451934, 11.67502) | 0.2717 |
| **comorbidities [3 or more]** | 2.770351 (0.4951477, 18.91574) | 0.2635 |
| **site [Hopkins]** | 183430700 (1.104490e-57, NA) | 0.9899 |
| **site [Thailand]** | 79575820 (2.875686e-21, 2.084527e+220) | 0.9904 |
