# Supplementary material for: COVID-19 symptom severity and duration among outpatients, July 2021-May 2023: The PROTECT observational study
Source: PLoS One. 2025 Feb 21;20(2):e0314518. doi: 10.1371/journal.pone.0314518 (PMC11844841; doi:10.1371/journal.pone.0314518)
Supplement: S2 Table — (DOCX) [file pone.0314518.s002.docx]

|  | **Coefficient (95% Confidence Interval)** | **p-value** |
| --- | --- | --- |
| **sex [Male]** | 1.7497778 (1.0632894, 2.879482) | 0.0277 |
| **age [>=45]** | 0.7337486 (0.4366092, 1.233110) | 0.2425 |
| **race [Black or African American]** | 0.7509173 (0.3108498, 1.813985) | 0.5244 |
| **race [Asian]** | 0.4117436 (0.1443231, 1.174676) | 0.0971 |
| **race [Other]** | 1.3821596 (0.3990668, 4.787081) | 0.6096 |
| **vaccination [2 or more doses]** | 0.7240638 (0.2883859, 1.817941) | 0.4918 |
| **comorbidities [1]** | 1.6335330 (0.8649836, 3.084949) | 0.1303 |
| **comorbidities [2]** | 0.8420408 (0.3635476, 1.950316) | 0.6883 |
| **comorbidities [3 or more]** | 0.8241843 (0.3358715, 2.022439) | 0.6729 |
| **site [Hopkins]** | 1.4906274 (0.6326114, 3.512377) | 0.3613 |
| **site [Thailand]** | 8.3450761 (2.4581387, 28.330498) | 0.0007 |
