# Supplementary material for: COVID-19 symptom severity and duration among outpatients, July 2021-May 2023: The PROTECT observational study
Source: PLoS One. 2025 Feb 21;20(2):e0314518. doi: 10.1371/journal.pone.0314518 (PMC11844841; doi:10.1371/journal.pone.0314518)
Supplement: Attachment — Submitted filename: PONE-D-24-20524.pdf [file pone.0314518.s005.pdf]

16 September 2024

The Editor

PLOS ONE
plosone@plos.org

Manuscript Number: PONE-D-24-20524

Manuscript Title: COVID-19 symptom severity and duration among outpatients, July 2021-May 2023: The PROTECT observational study

My assessment / comments are as follows:

1. Is the manuscript technically sound, and do the data support the conclusions?
   Yes

2. Has the statistical analysis been performed appropriately and rigorously?
   Yes, extensive and appropriate statistical analyses have been performed and reported.

3. Have the authors made all data underlying the findings in their manuscript fully available?
   Yes, de-identified data can be obtained from the corresponding author.

4. Is the manuscript presented in an intelligible fashion and written in standard English?
   Yes

5. Review comments to the Author:

   a. Page 2, Abstract:

      - Line 30: I suggest that the authors use "United States of America" instead of just United States.

      - Line 37: 9% Delta and 90% Omicron. What constituted the other 1%?

      - Lines 36, 43: Starting a sentence with a number (241 participants; 55% of SARS-CoV-2-positive) makes reading difficult. The sentence needs to be reconstructed or figures should be written out in full. This also occurs elsewhere in the manuscript.

   b. Page 5, Line 99: What was the reason / rationale for selecting this specific timeframe (June 15, 2021 to May 25, 2023)? Was this purely convenience sampling or was there any clinical relevance attached to the different periods at different sites?

   c. Pages 5 & 6, Lines 111, 112: Why was enrolment window different for cases collected during 2021 ("ten days if enrolled between July 15, 2021 to May 21, 2021")? Are these dates correct, since it is not in chronological order?

Fakulteit Geneeskunde en Gesondheidswetenskappe
Faculty of Medicine and Health Sciences
Prof Corena de Beer,
Chair: Postgraduate Program Committee / Senior Lecturer / Principal Medical Scientist
Division of Medical Virology, Department of Pathology, Clinical Building, Faculty of Medicine and Health Sciences,
Stellenbosch University, P O Box 241, CAPE TOWN, 7505, South Africa
Tel: +27 27 938 9453, 082 826 0453 / Fax: +27 21 938 9361, 086 621 3268 / e-mail: cdeb@sun.ac.za

d. Page 6, Lines 120, 121: What is the volume of blood collected with the Tasso SST blood collection device?

e. Page 9, Lines 184, 185, 195: Error! Reference source not found. Tis is also the case in several other places in the manuscript.

f. Page 10, Lines 199, 200: What type of vaccines were received by those individuals reporting that they had been vaccinated? Would you expect a difference in the number and severity of symptoms between recipients of mRNA, vector and protein subunit vaccines?

g. Page 17, Lines 326, 327: What measures were put in place to avoid or limit bias in the self-reporting symptoms? Is it possible that (recall and survivor, amongst other) bias could have been the reason for the absence of an association between symptom severity and any of the other parameters measured?

h. When comparing cohorts from vastly different geographical and demographical perspectives, such as the USA and Thailand, it should be justified why the authors think the two countries are comparable. Besides the predominance of different VOCs circulating in the USA and Thailand, there are additional factors that could have played a role, such as population densities and other cultural practices. Was this considered when selecting the sites?

i. Finally, as part of the Discussion, I would have liked to see a section on how the current study can contribute to respiratory infections and illnesses post-COVID-19 pandemic.

I found this manuscript insightful and of high quality, but feel that addressing the above questions will enhance the value of the research. It is a pity about the references not being found, as it made it difficult to properly evaluate the facts.

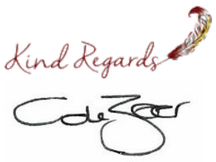

Prof Corena de Beer | PhD

Associate Professor | Principal Medical Scientist

Chair: Stellenbosch University, Post Graduate Programme Committee (Medical Virology);
Vice-chair: Research Ethics Committee - Biological and Environmental Safety
Chair: National Department of Health, National Task Force (WHO Global Polio Eradication)

Faculty of Medicine and Health Sciences | National Health Laboratory Service
Room 8002, Clinical Building, Francie van Zijl Drive, Tygerberg Campus | South Africa

Tel: +27 21 938 9453 | +27 82 826 0453; Fax: +27 21 938 9361 | 086 621 3268
cdeb@sun.ac.za | corena.debeer@nhls.ac.za
www.sun.ac.za | www.sun.ac.za/english/faculty/healthsciences/virology/Pages/Medical_Virology.aspx
