# Supplementary material for: COVID-19 symptom severity and duration among outpatients, July 2021-May 2023: The PROTECT observational study
Source: PLoS One. 2025 Feb 21;20(2):e0314518. doi: 10.1371/journal.pone.0314518 (PMC11844841; doi:10.1371/journal.pone.0314518)
Supplement: Attachment — Submitted filename: Plos One Response 18 July 2024.docx [file pone.0314518.s004.docx]

To: Editorial Manager, PLOS ONE

In response to the request from the Editorial Manager, PLOS ONE, regarding the point of contact on the core IRB (Advarra) approval letter. Per the Henry Jackson Foundation for the Advancement of Military Medicine (HJF) policy and contract with Advarra, all communications between the two entities are directed to the HJF Director of Regulatory Affairs, Maryanne Spevak. This is the reason why the protocol approval letter is addressed to Maryanne Spevak and not the principal investigator on the study.

In response to the Editorial Manager’s inquiry, I have also attached to our manuscript submission the protocol approval letter for the version of the protocol that lists Kristen Pettrone (myself) as the principal investigator on the study in addition to the approved protocol.

I hope this letter and the additional documents provided adequately address the Editorial Manager’s inquiry

Kind Regards,

Kristen Pettrone
