# Supplementary material for: COVID-19 symptom severity and duration among outpatients, July 2021-May 2023: The PROTECT observational study
Source: PLoS One. 2025 Feb 21;20(2):e0314518. doi: 10.1371/journal.pone.0314518 (PMC11844841; doi:10.1371/journal.pone.0314518)
Supplement: Attachment — Submitted filename: Response to Reviewers Plos_One review 22Oct24.docx [file pone.0314518.s006.docx]

**Manuscript Number:** PONE-D-24-20524

**Manuscript Title:** COVID-19 symptom severity and duration among outpatients, July 2021- May 2023: The PROTECT observational study

The authors thank the Editor and reviewers for their review and insightful comments. The authors provide the following responses to each of the Reviewer’s and Editor’s comments. A revised clean and track changes version of the manuscript is included with this resubmission.

**Reviewer #1 Comments**

1. Review comments to the Author:
   a. Page 2, Abstract:
      - Line 30: I suggest that the authors use “United States of America” instead of just United States.

Author response: This has been changed.

- Line 37: 9% Delta and 90% Omicron. What constituted the other 1%?

Author response: The one percent constitutes those that were not able to be identified by variant analysis in addition to the one participant who was infected with the Iota variant.

- Lines 36, 43: Starting a sentence with a number (241 participants; 55% of SARS- CoV-2-positive) makes reading difficult. The sentence needs to be reconstructed or figures should be written out in full. This also occurs elsewhere in the manuscript.

Author response: This has been corrected throughout the document.

b. Page 5, Line 99: What was the reason / rationale for selecting this specific timeframe (June 15, 2021 to May 25, 2023)? Was this purely convenience sampling or was there any clinical relevance attached to the different periods at different sites?

Author response: This timeframe was constructed from the funding availability and subsequently meeting enrollment targets at all sites.

c. Pages 5 & 6, Lines 111, 112: Why was enrolment window different for cases collected during 2021 (“ten days if enrolled between July 15, 2021 to May 21, 2021”)? Are these dates correct, since it is not in chronological order?

Author response: This 10 days refers to the enrollment window from symptom onset. Early in the study, we allowed participants with longer times from onset of symptoms to enrollment (10 days). Later in the study however, we restricted this to 5 days post symptoms onset to be eligible for enrollment to be more in line with the upcoming planned clinical trial. This has been clarified in the manuscript

d. Page 6, Lines 120, 121: What is the volume of blood collected with the Tasso SST blood collection device?

Author response: The Tasso SST collects from 250 to 500 µL per draw.

e. Page 9, Lines 184, 185, 195: Error! Reference source not found. Tis is also the case in several other places in the manuscript.

Author response: These were the links to the Tables and Figures in the document. These links have been removed.

f. Page 10, Lines 199, 200: What type of vaccines were received by those individuals reporting that they had been vaccinated? Would you expect a difference in the number and severity of symptoms between recipients of mRNA, vector and protein subunit vaccines?

Author response: Given the wide geographical spread of the study population and differences in vaccine availability in the respective countries, participants received predominantly mRNA and the available vector vaccines. While the authors did consider the impact of the different types of vaccines on symptom duration and severity, the numbers were too small in each category to be able to make any meaningful inferences.

g. Page 17, Lines 326, 327: What measures were put in place to avoid or limit bias in the self-reporting symptoms? Is it possible that (recall and survivor, amongst other) bias could have been the reason for the absence of an association between symptom severity and any of the other parameters measured?

Author response: The symptom questionnaire was delivered electronically to participants daily. To assist with adherence to survey completion, participants received daily reminders via phone or email to complete the survey. Participants who did not complete the survey electronically were either called daily and the survey was conducted via interview format or participants were reminded to complete on paper forms they had received at enrollment. Additionally, to further avoid recall bias, the online survey was only available for 24 hours. If the participant did not complete the daily survey in the 24 hour window, that survey was no longer accessible to the participant. As the target population for the study is outpatients diagnosed with COVID-19 who are early in their course of illness, we do not expect survival bias to unduly influence the study findings.

h. When comparing cohorts from vastly different geographical and demographical perspectives, such as the USA and Thailand, it should be justified why the authors think the two countries are comparable. Besides the predominance of different VOCs circulating in the USA and Thailand, there are additional factors that could have played a role, such as population densities and other cultural practices. Was this considered when selecting the sites?

Author response: The authors agree that there are limitations in considering the study population as a whole given the geographic diversity of the sites. The authors acknowledge that there are additional factors such as demographics, cultural practices, host responses and care seeking behaviors that may further contribute differences in results among the sites. We have attempted to take this into account by providing some study results and interpretations stratified by site. In choosing sites, the authors worked within existing study site relationships and chose sites able to implement an observational study in the setting of a pandemic.

i. Finally, as part of the Discussion, I would have liked to see a section on how the current study can contribute to respiratory infections and illnesses post-COVID-19 pandemic.

Author response: The authors have added additional information at the end of the discussion section on the contribution of this work to other known and novel respiratory viral infections.

**Editor Comments**

1. Line 62-65: "Understanding the physiologic changes, host biomarker responses, and early disease viral burden of acute respiratory infections (ARI) such as SARS-CoV-2 can provide valuable prognostic and predictive information allowing for rapid and timely diagnosis of patients, improved clinical management, and minimize disruptions to everyday life.": there are no references cited to support this claim. More references are suggested to be cited, with the following one as example (citing is optional):

Author response: References have been added to this section.

2. The RT-PCR for SARS-CoV-2 using ct value cannot provide viral load. Please avoid using "viral load" in this context as ct value is insufficient to provide viral load and PCR only detects RNA rather than viruses. Also, line 155, "N1 probe cycle threshold (Ct) value was used as an indicator of viral load." should be reworded ad this is inaccurate statement. Unless running standards, ct value can not predict viral load.

Author response: The authors have revised the line 155 in the manuscript to indicate that The N1 probe cycle threshold (Ct) value was used as an indicator of viral burden. The authors have also revised the results section and removed the reference to viral load and replaced with Ct value.

**Reviewer #2 Comments**

1. In line 26 (Introduction): While the paper mentions the emergence of new SARS-CoV-2 variants, there is insufficient exploration of the virological mechanisms behind immune evasion, viral mutations, or changes in symptomatology. Expanding this section to discuss the specific mutations in the Delta and Omicron variants that contribute to altered viral fitness, transmission, and immune evasion would strengthen the context for the study.

The authors appreciate the reviewer’s insightful comments. The authors have added additional information on the impact of mutations among VOC on therapeutic efficacy of interventions, disease patterns and testing (Lines 54-56).

2. In line 72 (Therapeutics Discussion): The paper briefly mentions the role of therapeutics in reducing COVID-19 severity but does not offer detailed, up-to-date information on the efficacy of antiviral treatments against the variants studied. Including recent findings on how treatments (e.g., monoclonal antibodies, antivirals) interact with variant-specific mutations would add depth to the study’s clinical implications.

Authors have updated the therapeutics discussion to reference approved therapies that reduce progression of severe disease in high-risk individuals. Authors have also added reference to the National Institutes of Health current COVID-19 Treatment Guidelines.

3. In line 150 (SARS-CoV-2 Sequencing): The variant sequencing methods are sound, but the study lacks detailed integration between the virological data and clinical outcomes. Providing more information on how specific mutations in Delta and Omicron variants (e.g., spike protein changes) align with observed differences in symptom severity and duration would significantly enhance the methodological clarity.

The authors appreciate the reviewer’s insightful comments and agree that impact of specific mutations on symptom manifestation and clinical outcomes would be useful however, we do not have enough sequencing data to be able to evaluate these correlations. The authors also feel a detailed evaluation of specific gene mutations and spike protein changes is outside the intended scope of this manuscript. The authors have added a section in the discussion section addressing differences in VOCs (genetics, host response, tissue tropism) and their potential impact on disease severity and symptom manifestation in ours and other studies.

4. In line 183 (Study Population Results): The study mentions co-infections with rhinovirus/enterovirus but does not explore how these co-infections might have affected SARS-CoV-2 symptomology or severity. Adding virological discussions on viral interference, co-infection dynamics, or immune modulation in the presence of multiple pathogens would provide a more complete understanding of the results.

Unfortunately, given the small numbers of SARS-CoV-2 positive patients with co-infections (n=5), we are not able to evaluate the effect of co-infections on the outcomes of interest. Lines 336-8 in the revised manuscript address this point.

5. In line 206 (Variant Testing): The paper describes the distribution of Delta and Omicron variants but lacks in-depth analysis of how variant-specific mutations contributed to the observed differences in symptom profiles. Discussing key mutations (e.g., in the spike protein or other viral components) that affect viral replication rates, immune evasion, or tissue tropism would improve the interpretation of these findings.

The authors have added a section in the discussion section addressing differences in VOCs (genetics, host response, tissue tropism) and their potential impact on disease severity and symptom manifestation in ours and other studies.

6. In line 218 (Symptomatology Results): Although the symptom data is presented for COVID-19 and ILI participants, there is no virological explanation for the observed differences. Including a discussion on how viral load kinetics, host immune response, and variant-specific replication dynamics might influence these differences would enrich the analysis.

The authors agree and have added a section in the discussion section addressing differences in VOCs (genetics, host response, tissue tropism) and their potential impact on disease severity and symptom manifestation in ours and other studies.

7. In line 253 (Symptom Prevalence by Variant): The result that Delta was associated with more eye and sensory symptoms than Omicron requires further virological context. The paper could benefit from exploring how viral mutations in Delta potentially alter tissue tropism (e.g., greater affinity for conjunctival cells or sensory neurons) compared to Omicron, thereby leading to these symptom differences.

The authors agree and have added a section in the discussion section addressing differences in VOCs (genetics, host response, tissue tropism) and their potential impact on disease severity and symptom manifestation in ours and other studies.

8. In line 264 (Viral Load Results): While viral load data is provided, the lack of correlation between viral load and symptom duration is not fully explored. A deeper discussion on variant-specific replication rates, immune responses (e.g., cytokine profiles), or viral persistence might explain why viral load did not show a significant association with symptom outcomes. Highlighting whether certain mutations in Delta or Omicron influence viral clearance rates could improve the understanding of the data.

Given the sampling time frames (Days 3, 7, 10, 14, 21, 28, and 90), we were not able to fully explore viral load and viral clearance of the VOCs in this study. The complex interplay between host immune responses, variant specific replication rates and viral clearance would indeed be a worthwhile exploration however an in-depth exploration of these interactions is beyond the scope of this manuscript.

9. In line 268 (Symptom Severity and Viral Load): The divergence in Ct values based on symptom severity is mentioned, but the underlying virological reasons are not discussed. The paper could be strengthened by addressing how early immune responses (e.g., interferon levels) or viral replication dynamics in specific tissues contribute to symptom severity in different SARS-CoV-2 variants.

The authors agree and have added a section in the discussion section addressing differences in VOCs (genetics, host response, tissue tropism) and their potential impact on disease severity and symptom manifestation in ours and other studies.

10. In line 283 (Multivariate Logistic Regression on Symptom Severity): The paper found no significant association between demographic factors and symptom severity, but does not delve into potential virological explanations. Exploring how host-pathogen interactions, such as the variability in immune response based on age, race, or comorbidities, might influence disease outcomes for different variants would add depth to the interpretation.

Authors have added context and potential explanations around the age and sex findings in the study. See lines 323 -324 and lines 338-340.

11. In line 295 (Cox Proportional Hazards for Symptom Duration): The shorter symptom duration in males lacks a virological or immunological explanation. Discussing sex-based differences in immune response (e.g., T-cell activation, antibody production) or differences in viral clearance rates would improve the scientific rationale behind this observation. Additionally, integrating data on how viral kinetics might differ between sexes for the studied variants would provide more robust conclusions.

Authors have added context and potential explanations around the age and sex findings in the study. See lines 323 -324 and lines 338-340.

12. In line 342 (Long-Term Symptoms): The study mentions the presence of long-term symptoms in participants, but does not explore the virological mechanisms that might contribute to post-acute COVID syndrome. A discussion on viral persistence, chronic inflammation, or immune dysregulation could better explain the high percentage of patients reporting symptoms after 90 days, particularly in relation to different SARS-CoV-2 variants.

The authors appreciate the reviewers comment on symptom persistence and post-acute COVID syndrome. The study, however, was not designed to address PACS and did not capture persistence of symptoms in detail beyond 28 days. As such, the authors feel that an in-depth exploration of viral mechanisms attributing to post-acute COVID syndrome is beyond the scope of this manuscript.

13. In line 355 (Symptom Fluctuations): The study addresses the fluctuating nature of symptoms but does not provide a virological explanation for these observations. Discussing how viral replication in different tissues or immune system reactivation might lead to fluctuating symptom patterns would offer a more comprehensive understanding of the disease course.

The manuscript does not attempt to characterize waxing and waning symptoms in our study population. The reference to the fluctuating symptoms is in the context of attempting to define an endpoint for which to model disease severity. The authors thank the reviewer for this comment but feel that providing virologic explanations for symptom fluctuation does not fit with the context of this reference in the manuscript.

14. In line 365 (Site Differences): Although site differences in symptom duration are noted, the paper could benefit from more detailed analysis on how environmental factors, regional viral strains, or differences in immune responses among populations could contribute to these differences. A virological comparison of viral fitness or immune escape between regions might also clarify the observed outcomes.

The authors thank the reviewer for this comment. Additional text (Lines 383-387) has been added addressing potential explanations for the geographic differences seen in symptom duration in our study.
